# Efficacy of Oxybrasion in the Treatment of Acne Vulgaris: A Preliminary Report

**DOI:** 10.3390/jcm11133824

**Published:** 2022-07-01

**Authors:** Karolina Chilicka, Aleksandra M. Rogowska, Renata Szyguła, Monika Rusztowicz, Danuta Nowicka

**Affiliations:** 1 Department of Health Sciences, Institute of Health Sciences, University of Opole, 45-040 Opole, Poland; renata.szygula@uni.opole.pl (R.S.); monika.rusztowicz@uni.opole.pl (M.R.); 2 Institute of Psychology, University of Opole, 45-052 Opole, Poland; arogowska@uni.opole.pl; 3 Department of Dermatology, Venereology and Allergology, Wrocław Medical University, 50-368 Wroclaw, Poland; danuta.nowicka@umed.wroc.pl

**Keywords:** acne vulgaris, oxybrasion, sebumeter, corneometer

## Abstract

There are many cosmetic methods to reduce skin eruptions in people with acne vulgaris. As oxybrasion is a safe method of exfoliating dead epidermis, our objective was to investigate its effectiveness in young women with acne vulgaris. The Global Acne Grading System (GAGS) and Derma Unit SSC 3 device (Sebumeter SM 815, Corneometer CM 825) were used to assess acne vulgaris and skin properties. Twenty-four women aged 19–21 years (*M* = 19.50, *SD* = 0.66) with diagnosed mild acne vulgaris and a high level of sebum (more than 100 μg/cm^2^) participated in the study. Women on any dermatological treatment within the last 12 months and/or hormonal contraception were excluded. Probands were randomly assigned to two equal groups. Group A (experimental) was oxybrased with 0.9% sodium chloride solution simultaneously with compressed oxygen. Group B (placebo) was the group treated with non-carbonated mineral water and oxygen from the device (not pure). A series of five treatments was performed at 10-day intervals. Skin parameters were measured before and 30 days after the end of treatment. As a result, in group A (experimental), skin hydration and GAGS improved, while sebum on the epidermis was reduced. No side effects were noted. We concluded that oxybrasion is effective in women with acne and safe, as it improved skin parameters; however, further research is needed.

## 1. Introduction

Microdermabrasion is one of the methods of superficial controlled exfoliation of the stratum corneum. This treatment is safe and noninvasive, and as a result, patients can return to their daily activities without any recovery period [1,2,3]. Three types of microdermabrasion can be distinguished: diamond microdermabrasion, corundum microdermabrasion, and oxygen microdermabrasion oxybrasion. Diamond microdermabrasion uses the vacuum generated by a device and a tip covered with diamond crystals to exfoliate the surface of the epidermis. Corundum microdermabrasion uses corundum as an abrasive medium, while oxybrasion uses a stream of 0.9% sodium chloride solution ejected from an injector under pressure. In the case of corundum or diamond microdermabrasion, the friction causes skin redness and a feeling of warmth on the client’s skin. During oxybrasion, the treatment area is cooled with the cold stream of saline, which reduces discomfort during exfoliation [4,5].

Oxybrasion is an exfoliating and cleansing treatment that stimulates and increases blood circulation. It stabilizes the functioning of the sebaceous glands, and also oxygenates and moisturizes the skin. This method can be combined with chemical exfoliation or intense pulsed light therapy. Oxybrasion exfoliates the stratum corneum and reduces seborrhea. The treatment is based on the use of a 0.9% sodium chloride solution and pure oxygen from a cylinder, which eliminates the environment of anaerobic bacteria, in turn contributing to better treatment effects and the reduction of skin inflammation.

Microdermabrasion treatments are recommended for people with acne scars, stretch marks, keratoconus, and excessive sebum secretion, as well as for those with mature skin and people suffering from acne [6,7,8].

Acne vulgaris is a chronic inflammatory skin disease, which is characterized by the appearance of skin eruptions such as comedones, papules, nodules, pustules, and seborrhea. In the course of this disease, there is also an excess of *Cutibacterium acnes* bacteria, which leads to inflammation. *C. acnes* is a gram-positive, polymorphic bacterium, which is mainly in a cylindrical form, and which does not produce spore forms. Additionally, it is devoid of motor organelles. *C. acnes* prefers anaerobic conditions, although it is partially oxygen tolerant. It produces large amounts of porphyrins, which may contribute to the destruction of keratinocytes [9].

Among the factors that make people predisposed to acne, apart from the presence of certain bacteria (*C. acnes*, *Staphylococcus aureus*, and *Staphylococcus epidermidis*), the following can be distinguished: increased sebum production, abnormal keratinization of the sebaceous canal, hormonal disorders, and genetic factors [10,11,12]. In people with severe acne, the first step is dermatological treatment, which is designed to reduce the number of nodules and cysts. If the acne is mild or moderate and the physician agrees to use cosmetology treatments, many different methods can be proposed to improve the quality of the patient’s skin. The aim of the study was to check whether the oxybrasion treatment will have a positive effect on the reduction in skin eruptions.

## 2. Materials and Methods

### 2.1. Study Design

A single-blind placebo study with follow-up analysis was conducted at the Institute of Health Sciences of the University of Opole, Poland from February to April 2021. The research was approved by the Human Research Ethics Committee of the Opole Medical School (No. KB/57/NOZ/2019) and conducted according to the principles of the Declaration of Helsinki. The study was registered at https://www.isrctn.com (No. ISRCTN 28257448) and accessed on 7 May 2020. All patients signed a written informed consent prior to the research, and agreed to make photographic documentation. The photos were taken before the first treatment and 30 days after the end of the treatment series. The participants were informed that they may withdraw from the examination at any time and without giving any reason.

**Figure 1:**
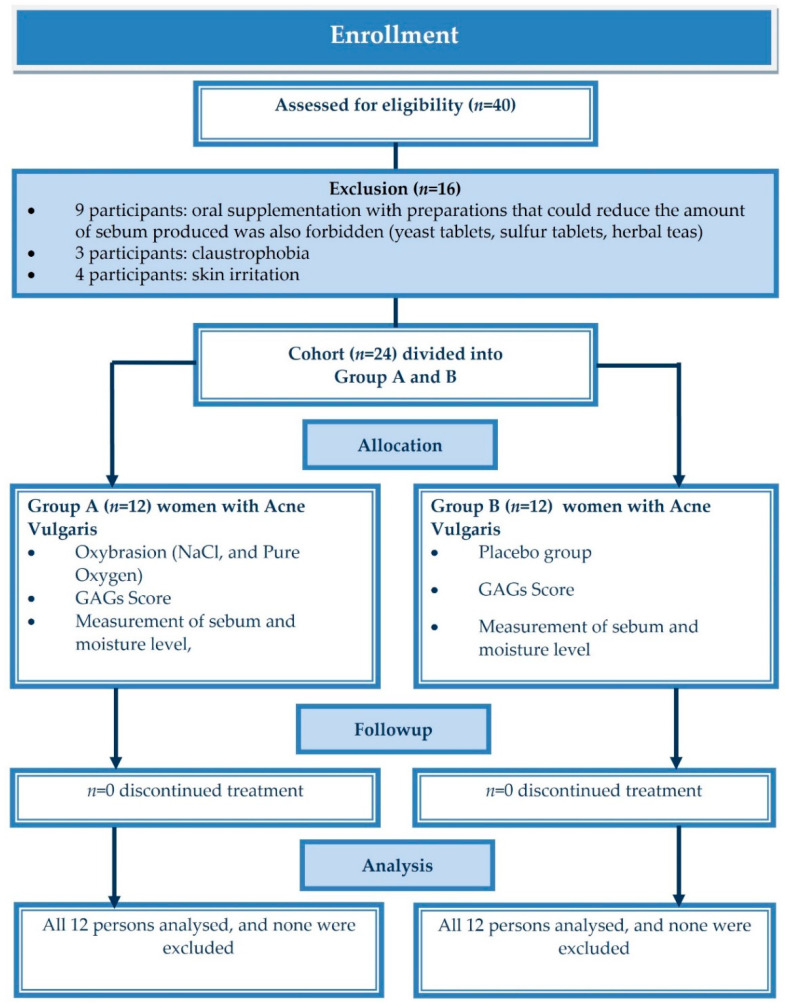
Patient follow in the study.

Initially, 40 people participated in the study, but only 16 met the exclusion criteria [oral supplementation with preparations that could reduce the amount of sebum produced was also forbidden (yeast tablets, sulfur tablets, herbal teas)—9 probants; claustrophobia—3 probants; skin irritation—4 probants]. The final study sample consisted of 24 women. The group was homogeneous due to the fact that no men entered the study. Twenty-four women, suffering from acne vulgaris, were divided into two groups. They were assigned to the groups on the basis of a random selection of envelopes containing a certain group designation (A or B). Group A (experimental) received the appropriate oxybrasion treatment using 0.9% sodium chloride solution and pure oxygen from a cylinder. Group B (placebo) had a “mock oxybrasion” procedure using still mineral water and oxygen from the compressor from the device (oxygen from the air). Patients from group B were not aware that they were a placebo group, and they were convinced that they were undergoing the procedure of oxybrasion. The process of inclusion of the patients is shown in Figure 1. All patients underwent a series of five treatments applied every 10 days. Any other cosmetic procedures, as well as the use of new cosmetics or sebum-regulating cosmetics, were forbidden during the study period. Only cosmetics such as micellar water and moisturizing creams were allowed.

### 2.2. Participants

A group of 24 women aged 19–21 (*M* = 19.50, *SD* = 0.66) who suffered from acne vulgaris participated in the study. All the tested participants were diagnosed with mild acne (100%) by the same rater dermatologist using the GAGS before and after a series of treatments in group A (experimental) and group B (placebo).

The mean duration of acne was 6 years (*M* = 5.79, *SD* = 0.83), with a range between 5 and 7 years. Inclusion criteria for this study were no dermatological treatment within 12 months, no current hormonal contraception, age 19–23 years, and having mild acne which was measured by the Global Acne Grading System (GAGS). The study had contraindications that made it impossible for some people to participate. These included: pregnancy, breastfeeding, epilepsy, claustrophobia, skin damage, taking oral medications within the last 3 months, taking isotretinoin within the last year, taking contraceptives, sun exposure after the procedure, tanned skin, skin cancers, psoriasis, eczema, viral infections (including herpes), bacterial and fungal skin diseases, skin irritation, active inflammation, active rosacea, psoriasis, atopic dermatitis, general illness, and a tendency to having sinusitis. Oral supplementation with preparations that could reduce the amount of sebum produced was also forbidden (e.g., yeast tablets, sulfur tablets, and herbal teas).

Group A (*n* = 12) and B (*n* = 12) included young adult women with a higher sebum level (more than 100 μg/cm^2^) and acne vulgaris. Acne, excessive seborrhea, blackheads, whiteheads, and papules were observed in the volunteers. Before and after the treatment series, the GAGS was used to determine the severity of acne and to check whether the treatments had a positive effect on the improvement of the skin condition of the patients.

### 2.3. Measures

#### 2.3.1. Acne Vulgaris

The severity of acne vulgaris was assessed using the GAGS, which was developed in 1997 by Doshi, Zaheer, and Stiller [13]. Acne, excessive seborrhea, blackheads, whiteheads, and papules were observed in the volunteers. The GAGS scale, which is used to determine the degree of advancement of acne and also to check whether the treatments had a positive effect on the improvement of the skin of the patients, was used before and after the treatment series. The scale includes the following areas: nose, cheeks, forehead, chin, as well as the back and chest. Each of them is assigned a number based on size: nose = 1; left cheek = 2; right cheek = 2; forehead = 2; chin = 1; and back and chest = 3. Depending on the degree of severity, each lesion is given a grade: no cutaneous conditions—0, comedones—1, papules—2, pustules—3, and nodules—4. The local score calculated for each area has the following formula: Local score = factor × Grade (0–4). The global score is composed of the sum of the local results: 1–18—mild acne, 19–30—moderate acne, 31–38—severe acne, and above 39—acne with a very severe course [14].

#### 2.3.2. Skin Parameters

Skin parameters were measured twice: before starting the tests (m0) and one month after the end of the last treatment (m1). The test subjects were asked to remove their face makeup the day before the measurements in the evening and not to apply any preparations to the face skin. The same applied to the morning care, with the difference that the patients could not remove make-up due to the fact that the parameters would be unreliable. Measurements were taken in the morning, with the test participants, after arriving in the room, acclimatizing for about 30 min. Room humidity was 40–50% and the temperature was 20–21 degrees C. The Derma Unit SCC 3 apparatus (Courge & Khazaka, Cologne, Germany) was used for the test. Skin hydration was tested with the Corneometer CM 825, while sebum was measured using the Sebumeter SM 815. The measurement points were as follows: forehead, nose, right and left cheeks, and chin.

### 2.4. Treatment Procedure (Intervention)

Before starting the treatment, in both groups A (experimental) and B (placebo), face makeup was removed using micellar fluid, and the skin was toned. The procedure was performed using a HEBE device. The patients’ ears were covered with cotton wool, their eyes were protected with cotton pads and rubber glasses, and a cosmetic cap was put on their hair. The treatment in group A (experimental) was the actual treatment of oxybrasion. Saline was used for the treatment, and it was administered with a manipulator at the parameters of 5–5.5 bar. The treatment started from the forehead, and then progressed to the area between the eyebrows, cheeks, nose, and chin. The manipulator was located approximately 0.5–1 cm from the face. The exfoliation procedure was performed for a total of 5 min. Then, after this time, the face was toned, dried with wipes, and blown with pure oxygen from the cylinder for 5 min using a manipulator. The treatment in group B (placebo) was performed with the aid of an applicator with still mineral water at the pressure of 5–5.5 bar. The scheme was the same as in the case of group A, but at the same time, oxygen was supplied from the manipulator with the mineral water, which was taken from the air using a compressor. The mineral water contained 213.40 mg/L of minerals in total: bicarbonate 121.06 mg/L; fluorides 0.07 mg/L; magnesium 5.37 mg/L; calcium 36.39 mg/L; and sodium 7.79 mg/L. After the treatment, groups A and B had a tonic and moisturizing cream applied to their faces. Each group had a series of 5 treatments 10 days apart.

For home care, it was recommended to wash the face twice a day using Cetaphil MD Dermoprotector (aqua, glycerin, hydrogenated polyisobutene, cetearyl alcohol, macadamia ternifolia seed oil/macadamia ternifolia nut oil, ceteareth-20, tocopheryl acetate, dimethicone, acrylates/C10-30 alkyl acrylate crosspolymer, benzyl alcohol, citric acid, farnesol, panthenol, phenoxyethanol, sodium hydroxide, stearoxytrimethylsilane, stearyl alcohol, FIL 0133.V02). After using the above-mentioned preparation, the patients were meant to apply Alantan Plus cream (20 mg allantoin and 50 mg dexpanthenol) as a 50% solution of panthenol in propylene glycol, lanolin, liquid paraffin, cetostearyl alcohol, ethyl parahydroxybenzoate, methyl parahydroxybenzoate, propyl parahydroxybenzoate, propyl parahydroxybenzoate, purified water, and polawax.

The patients were instructed not to use the swimming pool, or sauna, and to avoid exposure to natural and artificial radiation. They were meant to use photoprotection every day and not use any preparations other than Cetaphil MD Dermoprotector and Alantan Plus cream. During the entire series of treatments, and a month after its completion, it was forbidden to use any other cosmetic and aesthetic medicine treatments. Dermatological procedures for the duration of the study were also prohibited.

### 2.5. Statistical Analysis

Several statistics were checked to examine the effect of oxybrasion treatment on the faces of young adult women with acne. Firstly, descriptive statistics were performed to check the normality assumptions for all variables in the study, including age, years of acne disease, the severity of acne before and after treatment, moisturizing, and lubrication after treatment. Next, a Student’s *t*-test was conducted to examine differences between group A (experimental) and group B (placebo), for age, years of acne suffering, and severity of acne before and after the treatment with oxybrasion. Finally, the effect of oxybrasion on women’s faces was tested using a repeated measure two-way ANOVA. A post hoc test with Bonferroni correction was used to check significant differences. All statistics were conducted using JASP software for Windows: JASP Team, Version 0.14.1 (Computer software; Amsterdam, The Netherlands: Department of Psychological Methods, University of Amsterdam; 2020).

## 3. Results

### 3.1. Changes in Acne Severity after Oxybrasion Treatment

The preliminary analysis regards the parametric properties of the variables, using median, mean (*M*), standard deviation (*SD*), skewness, and kurtosis. Skewness and kurtosis ranged between −1.5 and +1.5, therefore parametric statistics were performed in the next steps of statistical analysis. The Student’s *t*-test was performed to examine differences between group A (experimental) and group B (placebo) in age, years of acne suffering, and acne severity diagnosis (Table 1). There were no group differences in age and diagnosis of acne before treatment. However, the groups differed slightly in years of acne suffering (*p* < 0.05), with a small effect size (Cohen’s *d* = −0.99). In addition, group A demonstrated significantly lower scores in acne severity than group B after treatment of oxybrasion (*p* < 0.001), with a large effect size (Cohen’s *d* = −7.04).

**Figure 2:**
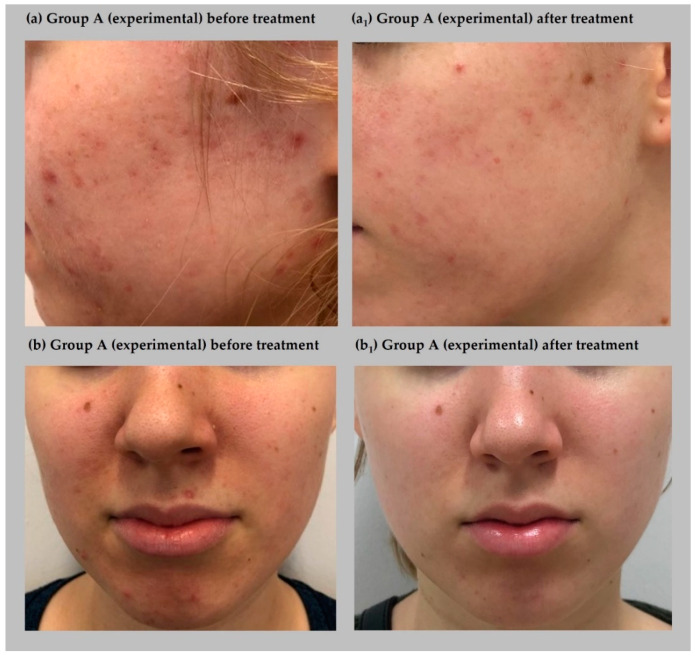
Effect of treatment on acne severity in two patients from group A (experimental) before (**a**,**b**) and after treatment (**a_1_**,**b_1_**).

**Figure 3:**
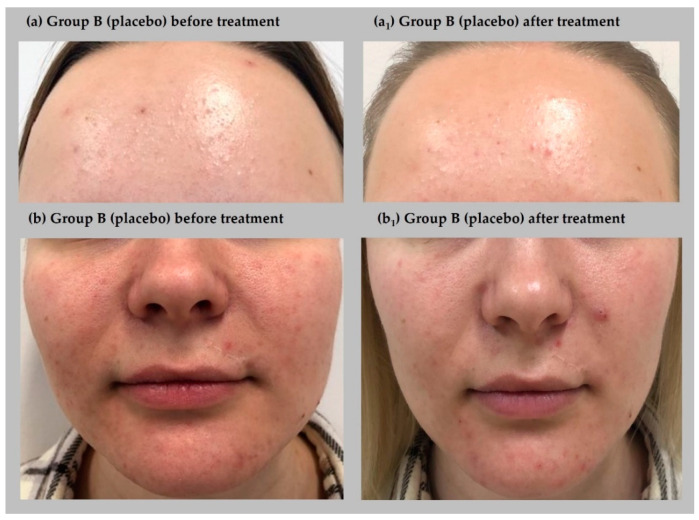
Effect of treatment on acne severity in two patients from group B (placebo) before (**a**,**b**) and after treatment (**a_1_**,**b_1_**).

A repeated measure two-way ANOVA was performed to examine the complex effect of oxybrasion on acne severity among women. The results indicated significant differences in acne severity (GAGS scores) before and after oxybrasion treatment, *F*(1, 22) = 531.14, *p* < 0.001, η^2^*_p_* = 0.96. The effect of group was also significant, *F*(1, 22) = 106.48, *p* < 0.001, η^2^*_p_* = 0.83, as well as the interaction effect between group and treatment, *F*(1, 22) = 176.33, *p* < 0.001, η^2^*_p_* = 0.95. The post hoc test with Bonferroni correction showed that groups A (experimental) and group B (placebo) did not differ significantly before treatment. Additionally, no differences were found between group A before treatment and group B after treatment. Acne severity was not changed significantly in group B before and after placebo treatment. However, a significant improvement was observed in group A regardless of acne severity before and after oxybrasion treatment. Figure 2 shows patients representing group A (experimental) while Figure 3 demonstrates participants from group B (placebo), both before and after treatment.

### 3.2. Changes in Facial Skin Moisture after Oxybrasion Treatment

A series of repeated measures two-way ANOVAs were performed for the forehead, nose, right cheek, left cheek, and chin to examine the effect of oxybrasion on moisturizing faces of acne patients (Table 2). The group effect was not significant for moisturizing each part of the face. In contrast, a significant effect (with a medium effect size) of oxybrasion treatment and interaction between group and treatment was found for the forehead, nose, right cheek, and left cheek. In contrast, oxybrasion treatment did not have any significant effect on the chin. Furthermore, a very small interaction effect was presented for the chins of women with acne.

### 3.3. Changes in Facial Skin Greasing after Oxybrasion Treatment

Repeated measures of two-way ANOVAs were conducted to examine oxybrasion effect on greasing of various parts of women’s faces (Table 3). The group effect was significant for the forehead, nose, right cheek, and left cheek, but insignificant for the chin. However, the effects of oxybrasion and interaction between treatment and group were significant for all parts of participants’ faces, with a medium or strong effect size.

## 4. Discussion

Our study showed that the procedure performed in group A (experimental) contributed to a significant reduction in the number of skin eruptions (GAGS scale) and improved skin appearance in patients in this group. In addition, group A demonstrated significantly lower scores in acne severity than group B after treatment of oxybrasion (*p* < 0.001), with a large effect size (Cohen’s *d* = −7.04) (Table 1). Table 3 shows how the oxybrasion affects greasing of various parts of women’s faces. The group effect was significant for the forehead, nose, right cheek, and left cheek, but insignificant for the chin. However, the effects of oxybrasion and interaction between treatment and group were significant for all parts of participants’ faces, with a medium or strong effect size. The study showed that a 0.9% sodium chloride solution exfoliated dead epidermis, and pure oxygen reduced skin eruptions in group A (experimental). *C. acnes* prefers anaerobic conditions, although it partially tolerates oxygen in temperatures of 36–37 °C.

Jarząbek et al. used oxybrasion on 27 healthy women; they examined sebum, skin hydration, pH, and transepidermal water loss (TEWL). Five treatments were performed every two weeks. The authors showed that the treatment had a positive effect on the reduction of the amount of sebum on the surface of the epidermis, in particular on the cheeks and nose. Oxybrasion increased skin hydration and decreased skin pH [4].

The most common method of microdermabrasion among researchers is diamond and corundum microdermabrasion. Chilicka et al. showed that treatments such as microdermabrasion and cavitation peeling had a positive effect on the condition of the skin in people with acne. There was a reduction in the number of skin eruptions, where as selected skin parameters, including sebum measurement, improved [15]. Fąk et al. performed the procedure for 16 women using aluminium oxide crystal microdermabrasion. The study examined whether the treatment would affect the level of hydration and lubrication of the epidermis. Statistically significant changes were observed on the cheeks and in the T-zone 30 min after the end of the procedure. There was a strong reduction of sebum all over the face; however, about an hour after the end of the treatment, this measurement began to return to the baseline. One hour after treatment, hydration started to gradually fall [16].

Kim et al. reported that diamond microdermabrasion was associated with an increase in TEWL immediately after the treatment, but after one day, it returned to the baseline value [17]. Abdel-Motaleb et al. showed morphological changes between the control group and the group treated using chemical peels and microdermabrasion. Salicylic acids and microdermabrasion significantly increased epidermal thickness, collagen fibres, and elastin thickness [18]. A study by El-Domyati et al. was conducted on 38 patients (four groups: acne scars, melasma, photoageing, and striae distensae). Eight microdermabrasion treatments were performed with a one-week interval. Punch biopsies were taken. Histometric analysis of epidermal thickness showed nonsignificant changes in all the groups. Collagen fibres with a more regular arrangement were detected in acne scars, and decreased melanization was also noted [19].

Kołodziejczak et al. used a combination of microdermabrasion and cavitation peeling in patients with seborrheic skin with acne punctata. Nine women had a series of six treatments with an interval of 10–14 days. Measurements such as skin hydration levels and sebumetric values were taken. In all patients, an improvement in skin sebum was observed. A significant improvement in skin hydration was seen in the chin area [19]. Scientists investigated 10 patients with a series of six microdermabrasion treatments with an interval of 14 days. One side of the face had two passes of microdermabrasion and the other side of the face had three passes. After the series of treatments, the sebum level was lower than baseline values. An increase in pH values was observed after finishing the series of microdermabrasion [20].

In the case of data concerning the effects of oxybrasion on acne-prone skin, there are no studies regarding this topic in the databases.

## 5. Study Limitations

This was a preliminary study, and therefore the research sample can be seen to be limited. In the future, we would like to increase the number of patients, as well as expand the group to include men, not only women.

## 6. Conclusions

Oxybrasion treatment using saline and pure oxygen from a cylinder significantly reduced the skin sebum level and increased the skin hydration level on the right part of the face. This method also reduced the number of skin eruptions in the study participants (group A). This was visible on the photographic documentation, and also due to the use of a dermatological scale for the determination of the degree of acne (GAGS). The oxybrasion treatment using 0.9% sodium chloride solutions and pure oxygen is a safe procedure with no side effects such as redness or skin irritation. It is a procedure that does not require the person undergoing it to be excluded from everyday functioning. However, it should be remembered that dermatological treatment cannot be replaced by cosmetological treatment.

## Figures and Tables

**Table 1:** Descriptive statistics.

Variable	Total Sample		Group A		Group B		*t*(22)	*p*	*d*
	*M*	*SD*	*M*	*SD*	*M*	*SD*			
Age	19.50	0.66	19.25	0.45	19.75	0.75	−1.97	0.062	−0.80
Years of acne	5.79	0.83	5.42	0.67	6.17	0.84	−2.43	0.024	−0.99
GAGS before	17.08	0.88	17.17	1.03	17.00	0.74	0.46	0.653	0.19
GAGS after	12.75	3.97	9.00	1.13	16.50	1.00	−17.23	<0.001	−7.04

Note. Group A = experimental, Group B = placebo, GAGS = Global Acne Grading System, *M* = mean, *SD* = standard deviation, *t* = Student’s *t*-test, *p* = significance level, *d* = Cohen’s *d* effect size.

**Table 2:** The repeated measures one-way ANOVA for moisturizing using Corneometer CM825 (g/m^2^).

Moisturizing	Group A		Group B		Effect	*F*(1, 22)	*p*	η^2^*_p_*
	*M*	*SD*	*M*	*SD*				
**Forehead**					G	2.61	0.120	0.11
Before	41.69	5.63	41.87	6.30	T	44.26	<0.001	0.67
After	49.81	5.44	42.18	6.01	T × G	37.86	<0.001	0.63
**Nose**					G	1.27	0.272	0.06
Before	41.93	6.92	43.58	6.45	T	30.87	<0.001	0.58
After	51.54	5.15	44.50	6.54	T × G	21.05	<0.001	0.49
**Right Cheek**					G	0.69	0.416	0.03
Before	41.61	8.54	41.97	8.07	T	43.92	<0.001	0.67
After	49.03	7.41	43.34	8.09	T × G	20.74	<0.001	0.49
**Left Cheek**					G	1.23	0.279	0.05
Before	43.72	8.03	43.63	7.89	T	20.57	<0.001	0.48
After	50.77	7.97	44.23	6.33	T × G	14.62	<0.001	0.40
**Chin**					G	0.09	0.766	0.00
Before	38.98	4.62	40.84	5.58	T	3.61	0.071	0.14
After	41.30	4.57	40.71	6.34	T × G	4.54	0.045	0.17

Note. Group A = experimental, Group B = placebo, G = group, T = treatment. *M* = mean, *SD* = standard deviation, *F* = Fisher’s *F*-test, *p* = significance level, η^2^*_p_* = partial eta-square effect size.

**Table 3:** The repeated measures one-way ANOVA for greasing using Sebumeter SM815 (μg/cm^2^).

Greasing	Group A		Group B		Effect	*F*(1, 22)	*p*	η^2^*_p_*
	*M*	*SD*	*M*	*SD*				
**Forehead**					G	12.8	0.002	0.37
Before	187.08	30.73	191.08	28.86	T	146.8	<0.001	0.87
After	115.08	24.92	185.75	21.47	T × G	109.1	<0.001	0.83
**Nose**					G	9.1	0.006	0.29
Before	180.08	23.17	180.42	22.70	T	87.4	<0.001	0.80
After	124.42	16.42	174.67	24.93	T × G	57.7	<0.001	0.72
**Right Cheek**					G	7.4	0.013	0.25
Before	182.75	27.74	178.33	26.05	T	130.2	<0.001	0.86
After	112.83	18.91	170.83	28.27	T × G	84.6	<0.001	0.79
**Left Cheek**					G	9.1	0.006	0.29
Before	180.92	24.35	181.25	23.03	T	58.3	<0.001	0.73
After	125.00	13.12	171.42	24.37	T × G	28.6	<0.001	0.57
**Chin**					G	1.0	0.334	0.04
Before	180.17	19.63	165.92	25.34	T	78.0	<0.001	0.78
After	129.75	12.62	160.00	25.15	T × G	48.7	<0.001	0.69

Note. Group A = experimental, Group B = placebo, G = group, T = treatment. *M* = mean, *SD* = standard deviation, *F* = Fisher’s *F*-test, *p* = significance level, η^2^*_p_* = partial eta-square effect size.

## Data Availability

Not applicable.
